# A Compact Dual Band MIMO Dielectric Resonator Antenna with Improved Performance for mm-Wave Applications

**DOI:** 10.3390/s22135056

**Published:** 2022-07-05

**Authors:** Meshari D. Alanazi, Salam K. Khamas

**Affiliations:** Communications Research Group, Department of Electronic and Electrical Engineering, The University of Sheffield, Mappin Street, Sheffield S1 3JD, UK; s.khamas@sheffield.ac.uk

**Keywords:** MIMO, high isolation, dielectric resonator antenna, compact size, 5G

## Abstract

A compact multiple-input-multiple-output (MIMO) dielectric resonator antenna (DRA) that is suitable for internet of things (IoT) sensor networks is proposed with reduced coupling between elements. Two rectangular-shaped DRAs have been placed on the opposite sides of a Rogers substrate and each is fed using a coplanar waveguide (CPW) feed with slots etched in a dedicated metal ground plane that is located under the DRA. Moreover, locating the elements at the opposite sides of the substrate has improved the isolation by 27 dB without the need to incorporate additional complex structures, which has reduced the overall antenna size. Furthermore, a dual band operation is achieved since each antenna resonates at two frequencies: 28 GHz and 38 GHz with respective impedance matching bandwidths of 18% and 13%. As a result, the corresponding data rates are also increased independently. In addition to the advantages of improved isolation, compact size and dual band operation, the proposed configuration offers a diversity gain (DG), envelope correlation coefficient (ECC) and channel capacity loss (CCL) of 9.98 dB, 0.007, 0.06 bits/s/Hz over the desired bands, respectively. A prototype has been built with good agreement between simulated and measured results.

## 1. Introduction

Dielectric resonator antennas have been used in a wide range of microwave related applications due to their well-known benefits such as design flexibility, low loss, and high radiation efficiency [1,2]. Modern wireless communications such as video streaming and multimedia applications demand higher data rates. A solution that was proposed to cater for such applications is to utilize a MIMO system with improved data rate for transmitting and receiving applications. However, a major challenge in the MIMO antenna setup is the mutual coupling between multiple antennas that are placed in the proximity of each other. Mutual coupling causes significant impedance mismatch, high correlation and impacts the radiation patten. Therefore, compact MIMO antennas with improved isolation are needed in modern wireless applications [3].

Additionally, multi-band antennas have received considerable attention as they are capable of operating in multiple applications simultaneously and can replace several single band antennas that are needed otherwise. In order to meet the high data rates demanded by modern communications systems, multiband MIMO antennas have been proposed [4]. However, developing MIMO antenna structures with a multiband operation represents another challenge due to the space constraints in particular for applications that require compact structures. Yet, when the frequency is increased, the wavelength becomes shorter and so does the physical size of the MIMO antenna. This makes it more complex to implement techniques that improve the performance of the antenna such as adding air-filled holes or a dielectric wall.

Several designs of 5G-and-beyond MIMO antennas working at a frequency range of 26 to 40 GHz have been proposed [5,6,7,8,9,10]. For example, loading a dipole antenna with a metamaterial surface was suggested for increasing the end-fire gain [5]. Similarly, a superstrate was used to improve the bandwidth and gain of a mm-wave microstrip antenna [6]. Even though such antennas exhibit high gain, the incorporation of the superstrate has increased the size and created mechanical complications. To address these issues, a patch antenna array of four elements with a defected ground was proposed and utilised in a MIMO configuration to obtain broadband and high gain simultaneously [7]. The impact of a defected ground plane was further investigated in [8] for the design of a 5G MIMO antenna with ultra-broadband and high gain. A MIMO antenna consists of eight H-shaped radiating elements has been proposed for mm-wave applications [9].

On the other hand, DRAs offer higher gains and wider bandwidths, which makes them suitable to be used in mm-wave applications while avoiding the conductor losses of patch antennas [10]. In earlier research studies, approaches such as varying the placement [11], polarization [12], and main-beam direction [13] have been implemented to decrease the mutual coupling between the DRAs. In addition, electromagnetic band gap (EBG) structures [14], frequency selective surface (FSS) walls [15,16], meta surface shields [17], metamaterial polarization-rotator walls [18], partially reflecting surfaces [19,20], and many more structures have been employed to reduce the mutual coupling between DRAs. However, all those configurations share a limitation of being complex and bulky. In order to reduce the complexity of the decoupling structure, metal strips were printed on the top DRA surface [10], metallic vias were implanted [21], metallic strips were added on the ground plane side [22] or both metallic strips and slots were utilized [23]. However, at mm-wave frequencies, approaches such as printing on the DRA top or drilling the DRA sides can represent major challenges and require precise machining tools that are complex and costly due to the small DRA physical dimensions at such frequencies. The proposed design offers simple and low-cost approach to achieve a high isolation between mm-wave DRAs in a compact size MIMO system. Besides, to the best of the authors’ knowledge, the achieved transmission coefficient, $S_{21}$, is lower than those reported in earlier studies. Furthermore, high broadside gain and wide bandwidth are achieved at the two operating frequency bands with low envelope correlation and high diversity gain. Therefore, the presented configuration addresses well-known limitations with respect to the structure and performance of mm-wave MIMO DRA systems. Furthermore, a dual band operation has not been reported earlier for MIMO DRAs. Each of the achieved bandwidth is ∼5 GHz at the 28 GHz and 38 GHz frequency bands that are widely used for Internet of Things (IoT) applications [24,25,26]. Additionally, a high date rate and compact size makes the proposed antenna suitable for miniature 5G devices.

## 2. Proposed Configuration

### 2.1. Rectangular DRA

**Figure 1:**
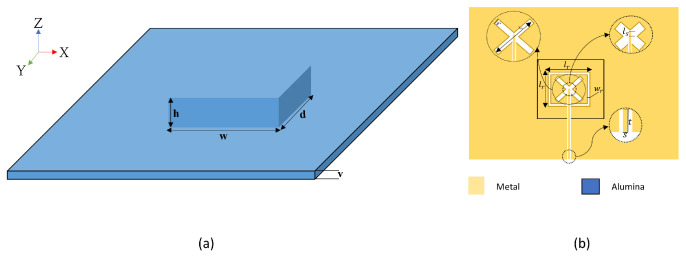
(**a**) 3D printed Alumina rectangular DRA and substrate (**b**) feeding structure.

**Figure 2:**
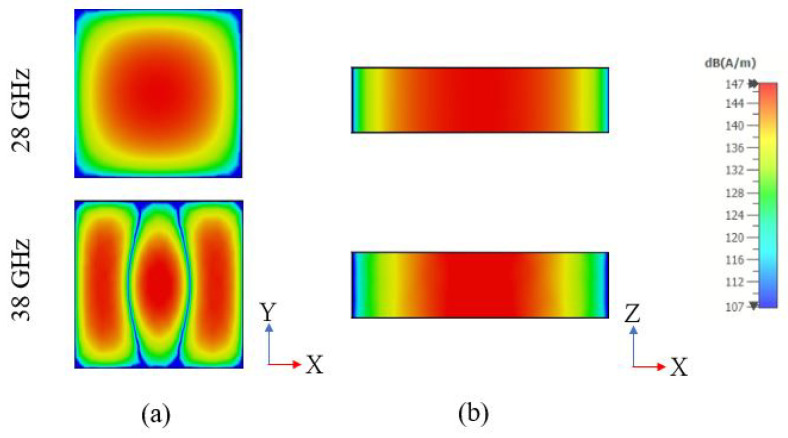
Fields distribution of the excited modes $TE_{111}$ at 28 GHz and $TE_{311}$ at 38 GHz (**a**) xy plane; (**b**) xz plane.

The RDRA is excited using coplanar waveguide (CPW) with a combination of cross and square slots as illustrated in Figure 1b. For the chosen DRA’s width, *w*, depth, *d*, and height, *h*, the modes’ resonance frequencies have been calculated by employing the well-known dielectric waveguide model [21], i.e.,

(1) $$k_{y}=\frac{n\pi }{w},k_{z}=\frac{m\pi }{2d},k_{x}^{2}+k_{y}^{2}+k_{z}^{2}=\varepsilon_{r}k_{0}^{2}$$

(2) $$k_{x}\tan\left(\frac{k_{x}h}{2}\right)=\sqrt{\left(\left(\varepsilon_{r1}-1\right)k_{0}^{2}-k_{x}^{2}\right)},f_{0}=\frac{c}{2\pi \varepsilon_{r}}\sqrt{\left(k_{x}^{2}+k_{y}^{2}+k_{z}^{2}\right)}$$

where $k_{x},k_{y}$, and $k_{z}$ are the wave numbers. For a rectangular DRA above a ground plane, the transverse electric, TE, modes are excited. With the aid of Equations (1) and (2), the required DRA dimensions have been calculated so that the fundamental and higher order modes of $TE_{111}$ and $TE_{311}$ can be excited at 28 GHz and 38 GHz, respectively. In addition, the DRA width and depth have been chosen so that a low DRA profile is achieved. Therefore, the calculated dimensions are *w* = *d* = 5 mm, and *h* = 1 mm, which offers a compact DRA with a profile that is lower than those reported in the literature. The magnetic field distribution of each mode is illustrated in Figure 2 using the CST Eigenmode solver, which is in line with the expected resonance modes using the dielectric wave guide model.

The slots’ dimensions need to be expressed in terms of the effective wavelength, $\lambda_{eff}$, which can be calculated as $\lambda_{eff}$ = $\lambda_{0}$/$\sqrt{\varepsilon_{eff}}$ and the effective permittivity is given by [27]:

(3) $$\varepsilon_{eff}\approx \frac{\varepsilon_{s}\varepsilon_{r}\left(t+h\right)}{\left(\varepsilon_{s}h+\varepsilon_{r}t\right)}$$

In Equation (3), $\varepsilon_{s}$, and *t* represent the permittivity and height of the Rogers substrate, respectively, and $\varepsilon_{r}$, and *h* represent the permittivity and height of the DRA, respectively. In this design, a combination of cross and square slots is utilized to excited two resonance modes and hence facilitates the dual band operation. The dielectric constant and height of the Rogers substrate are 2.3 and 0.25 mm, respectively. Therefore, the effective wavelengths have been calculated as 4.5 and 3.3 mm at 28 and 38 GHz, respectively. The cross slot has identical arms’ length and width of $l_{c}$ and $w_{c}$, respectively. The additional square slot was designed with a respective side’s length and arm width of $l_{r}$ and $w_{r}$. The PCB fabrication approach was followed to fabricate the feeding network using coplanar waveguide (CPW). The CPW feeding line incorporates a matching stub with a length of $l_{s}$ that is adjusted for optimum matching.

### 2.2. MIMO Configuration

**Figure 3:**
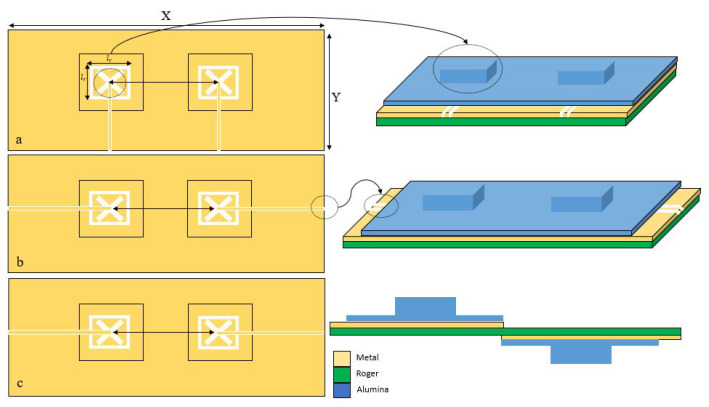
The MIMO RDRA configurations (**a**) Initial design with parallel CPW feeding lines (**b**) Intermediate design with collinear CPW feeding line (**c**) Final design with DRAs at opposite sides of the Rogers substrate and collinear CPW feeding lines.

As mentioned earlier, the proposed configuration involves two identical integrated DRAs that are placed on two separate feed networks printed on a Rogers 5881 substrate with thickness of 0.250 mm, relative permittivity of 2.3 and loss tangent, δ, of 0.0007. As illustrated in Figure 1, each integrated DRA is placed above one of the two feed networks that involve slotted ground planes. In order to simplify the antenna’s assembly, each DRA was printed on top of an Alumina substrate that has a thickness of *v* = 0.2 mm. The 3D printed Alumina DRA and substrate is illustrated in Figure 1a and will be referred to as an integrated DRA throughout this article.The evolution of the proposed design is demonstrated in Figure 3. The initial configuration of Figure 3a illustrates two integrated DRAs that are mounted on the same side of the Rogers substrate and the two CPW feeding lines are in parallel to each other. To minimise the coupling between the antennas, the configuration in Figure 3b was considered in which the CPW feeding lines’ orientation was modified so that they are collinear to each other and fed from the opposite sides of the substrate since one CPW feeding line has been rotated clockwise by 90° and the other has been rotated counter-clockwise by 90°. For further reductions of coupling, the two integrated DRAs and feed structures were placed on opposite sides of the Rogers substrate as demonstrated in Figure 3c. It should be noted that the recommended distance between the DRAs’ centres is $0.5\lambda_{0}$ [28]. The configuration’s parameters are listed in Table 1.

### 2.3. Surface Currents

**Figure 4:**
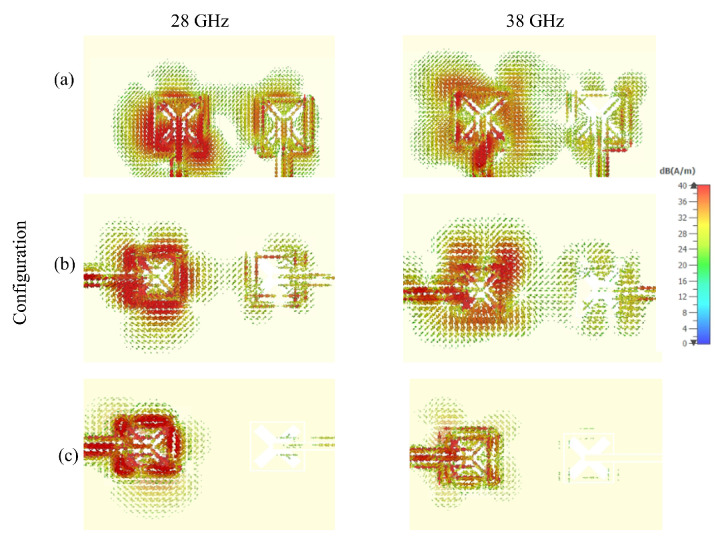
Surface current of the proposed MIMO antenna (**a**) Initial design with parallel CPW feeding lines (**b**) Intermediate design with collinear CPW feeding line (**c**) Final design with DRAs at opposite sides of the Rogers substrate and collinear CPW feeding lines.

The simulated current distributions at 28 GHz and 38 GHz have been studied by connecting a source to one DRA while the other DRA was parasitic and terminated by a 50 Ω lumped load. It should be noted that the current distributions in Figure 4a–c correspond to the configurations of Figure 3a–c, respectively. It is evident from Figure 4a that a noticeable current exists on the parasitic DRA’s feed, which indicates a strong mutual coupling since the two DRAs are close to each other and the feeding CPW lines are in parallel. Therefore, it can be concluded that such arrangement of DRAs and feed networks exhibit mutual coupling that can significantly affects the performance. On the other hand, a weaker current on the feed of the parasitic DRA can be observed in Figure 4b owing to the increased distance between the collinear CPW feeding lines in the configuration of Figure 3b. In addition, it is evident from Figure 4c that a rather weak current exists on the parasitic DRA’s feed when the antennas are placed on opposite sides of the Rogers substrate as in Figure 3c, which demonstrates a considerably reduced coupling and interference between the two integrated DRAs.

## 3. Results and Discussion

### 3.1. Performance of Single and MIMO DRAs

**Figure 5:**
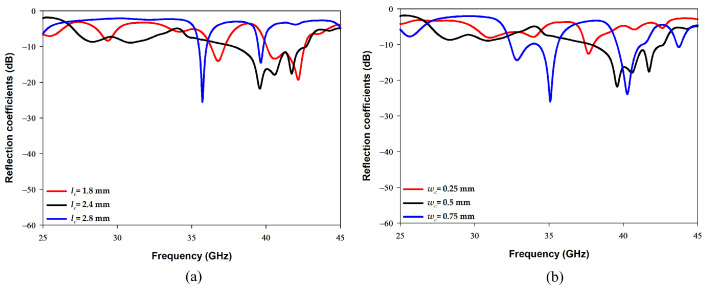
Effects of the cross-slot dimensions on the reflection coefficient (**a**) $L_{c}$ (**b**) $w_{c}$.

**Figure 6:**
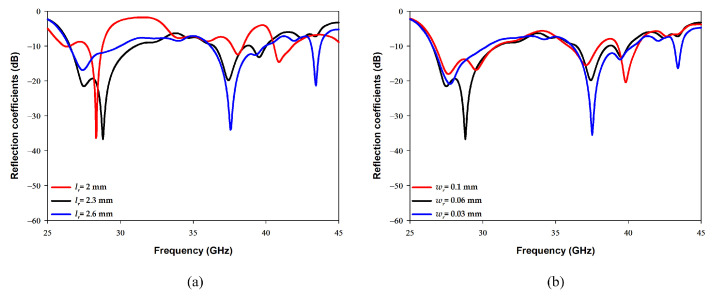
Effects of the square slot’s dimensions on the reflection coefficient (**a**) $L_{r}$ (**b**) $w_{r}$.

Figure 5a illustrates the effect of changing the cross-slot arm’s length, $l_{c}$, on exciting the required DRA resonance modes when the arm width was fixed at 0.5 mm. From these results it can be noted that the higher order mode is strongly excited when $l_{c}$ = 2.4 mm, which corresponds to ∼0.7$\lambda_{eff}$ at 38 GHz. On the other hand, Figure 5b demonstrates that a slot width of 0.5 mm is required to maintain the achieved resonance at 38 GHz. As mentioned earlier, a square slot has been added to excite the fundamental resonance mode $TE_{111}$ at 28 GHz while maintaining the excited higher order mode at 38 GHz. The optimized square slot dimensions are illustrated in Figure 6, where it can be noted that having an arm’s length of $l_{r}$ = 2.3 mm and width of $w_{r}$ = 0.06 mm provided the required dual band performance. It should be noted that $l_{r}$ corresponds to ∼0.5$\lambda_{eff}$ at 28 GHz. In addition, it can be noted from Figure 6a that by adding the square slot, the bandwidth of the upper band has also been increased by 5%. It is worth noting that a stub length of $l_{s}$ = 0.1 mm has provided an optimum matching. The resulting impedance bandwidths for the lower and upper bands are 18% and 13%, respectively. The achieved bandwidth is ∼5 GHz over each of the frequency bands of 28 GHz and 38 GHz, which results in an antenna configuration that is suitable for sensing, IoT and tracking applications.

**Figure 7:**
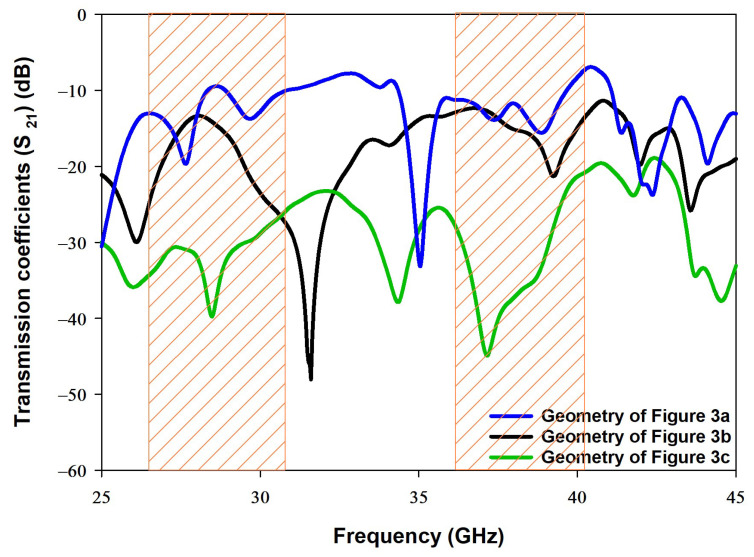
Simulated transmission coefficients for the configurations presented in Figure 3.

Furthermore, the impacts of altering the arrangement of the DRAs on the transmission coefficient, $S_{21}$, has been investigated for the three configurations of Figure 3, where it has been observed that altering the DRAs’ arrangement can significantly improve the isolation between elements. These results are demonstrated in Figure 7 in which the shaded areas indicate the frequency ranges over which impedance matching is achieved. The $S_{21}$ graph that corresponds to Figure 3a, offers transmission coefficients of −12 dB and −9.5 dB at 28 GHz and 38 GHz, respectively. This demonstrates that the upper frequency band has a stronger coupling between the DRAs, which may be attributed to the increased DRAs electrical size at 38 GHz. Furthermore, the results also indicate a modest isolation between the two DRAs since the parallel CPW feeding lines are close to each other. In addition, the smaller separation between the CPW feeding lines implies that, physically, the SMA will not fit comfortably, and hence it will affect the performance of any prototype.

On the other hand, the $S_{21}$ curve that corresponds to Figure 3b offers a slightly lower isolation of −14 dB at 38 GHz. As mentioned earlier, the proposed configuration of Figure 3c provides a simple and compact structure in which one DRA, and its feed network, are kept at the top side of the Rogers substrate and the other DRA and feed network are placed at the lower side of the same substrate. As a result, the $S_{21}$ has been reduced by 27 dB at 38 GHz compared to the case when the two DRAs are mounted at the same surface of the Rogers substrate. Therefore, reduced $S_{21}$ of −36 dB and −41 dB have been achieved at 28 GHz and 38 GHz, respectively. It is evident that the $S_{21}$ results of configuration of Figure 3c illustrates a considerably improved isolation. Another significant difference between the configurations is that a common ground plane was used in Figure 3a,b, whereas two separate ground planes were used in Figure 3c albeit with the same Roger substrate in all configurations. Therefore, the presence of the two ground planes on opposite sides of the Roger substrate has also contributed to the achieved in $S_{21}$ since they effectively act as a metal surface between the two DRAs.

### 3.2. Experimental Verification

**Figure 8:**
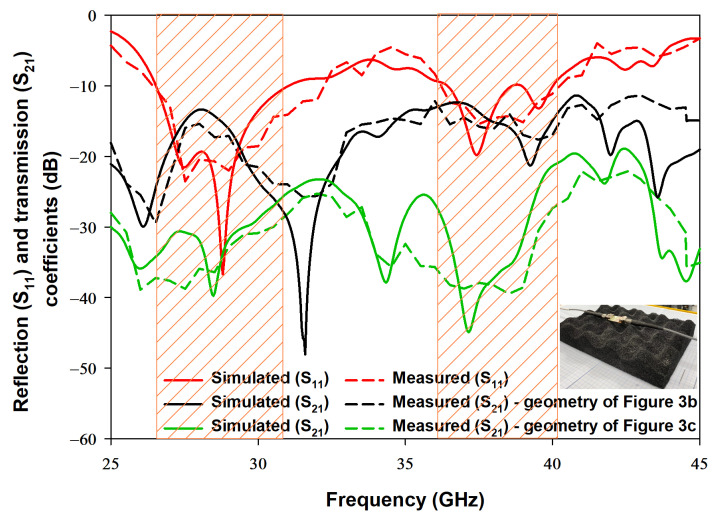
Simulated and measured S-parameters of the configurations in Figure 3b,c losses.

The proposed configurations in Figure 3b,c have been fabricated and measured using the E5071C mm-wave vector network analyser to measure the s-parameters through a 2.8 mm, 50 Ω, coaxial cable [29]. Moreover, a 2.8 mm SMA was used between the coaxial cable and the CPW feeding structure. The return losses are presented in Figure 8, where it is evident that the simulated and measured impedance bandwidths are in close agreement with each other. It is worth noting that the measured resonance frequencies, i.e., frequency points with minimum $S_{11}$, are 28.8 GHz and 37.61 GHz which are in good agreement with the dielectric waveguide mode calculation of Equation (2). It should be noted that the return losses are presented for the configuration of Figure 3c only as they are identical to those of the configuration in Figure 3b. The respective simulated and measured impedance bandwidth are 18% and 19.5% for the lower band and 13% and 15% for the upper band. The transmission coefficient’s results are also presented in Figure 8. For the configuration of Figure 3b, the respective simulated and measured transmission coefficients are −13 dB and −14.5 dB for the lower band, and −14 dB and −15.1 dB for the upper band. On the other hand, when the DRAs and their feed networks, are located at the opposite of the Rogers substrate, as in the geometry of Figure 3c, the respective simulated and measured transmission coefficients are −36 dB and −39 dB for the lower band, and −41 dB and −44 dB for the upper band. The simulated and measured transmission coefficients are in close agreement with each other. As expected, the transmission coefficient that corresponds to the configuration of Figure 3c is much lower than that of the counterpart in Figure 3b, which results in less interference and coupling between the DRAs in the proposed configuration.

**Figure 9:**
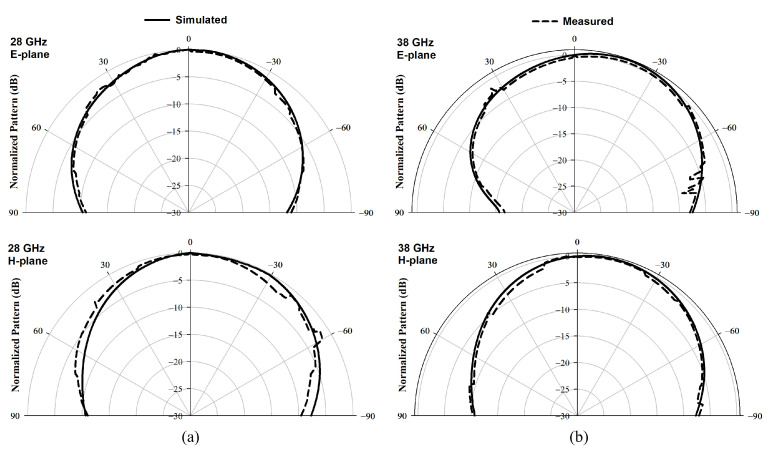
Radiation patterns of proposed MIMO configuration (**a**) E-plane (**b**) H-plane at 28 GHz and 38 GHz.

**Figure 10:**
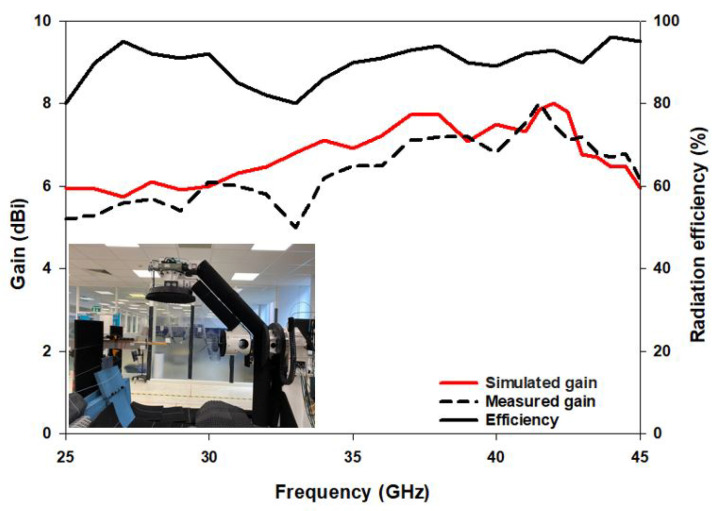
The broadside gain and simulated efficiency.

The spherical near-field mm-wave measurement system (SNF-FIX-1.0) was used to measure radiation pattern and the gain [29]. The arm of the SNF-FIX-1.0 spherical system is restricted to rotating across the upper hemisphere to cover the elevation angle’s range of θ = −90° to θ = 90°. The simulated and measured normalized broadside radiation patterns are presented in Figure 9 for the E and H-planes at 28 GHz and 38 GHz. Good agreement can be observed between simulated and measured radiation patterns. The measured gain of the DRA, $G_{\mathrm{DRA}(\mathrm{dB})}$, has been determined using the following Equation [30].

(4) $$G_{\mathrm{DRA}(\mathrm{dB})}=G_{\mathrm{Horn}(\mathrm{dB})}+10\log_{10}\frac{P_{\mathrm{DRA}}}{P_{\mathrm{Horn}}}$$

where $G_{\mathrm{Horn}}$(dB) denotes the gain of the reference mm-wave horn antenna, $P_{\mathrm{DRA}}$ is the power received by the DRA and $P_{\mathrm{Horn}}$ is the power transmitted by the horn antenna. The respective simulated and measured gains are 6.2 dBi and 5.8 dBi at 28 GHz, and 7.57 dBi and 7 dBi at 38 GHz as illustrated in in Figure 10 with close agreement between simulations and measurements. The increased gain at 38 GHz can be attributed to the excitation of the higher order DRA mode $TE_{311}$ at this frequency compared to the excitation of the lower order mode $TE_{111}$ at 28 GHz. In addition, the simulated efficiency is 90% over the operating frequency range as demonstrated in Figure 10, which is expected from the DRA due to the absence of ohmic and surface wave losses. There is a marginal discrepancy between simulated and measured results owing to fabrication errors and experimental tolerance. However, the discrepancies are more notable in the case of measuring the transmission coefficient for the configuration of Figure 3c, which can be attributed to a possible misalignment between two DRAs when they are placed on the opposite sides of the same substrate.

## 4. Performance of the MIMO Antenna

The characteristics of the MIMO antenna, particularly, the envelope correlation coefficient, the channel capacity loss, diversity gain, total active reflection coefficient, the mean effective gain and the multiplexing efficiency are evaluated in this section.

### 4.1. Envelope Correlation Coefficient

**Figure 11:**
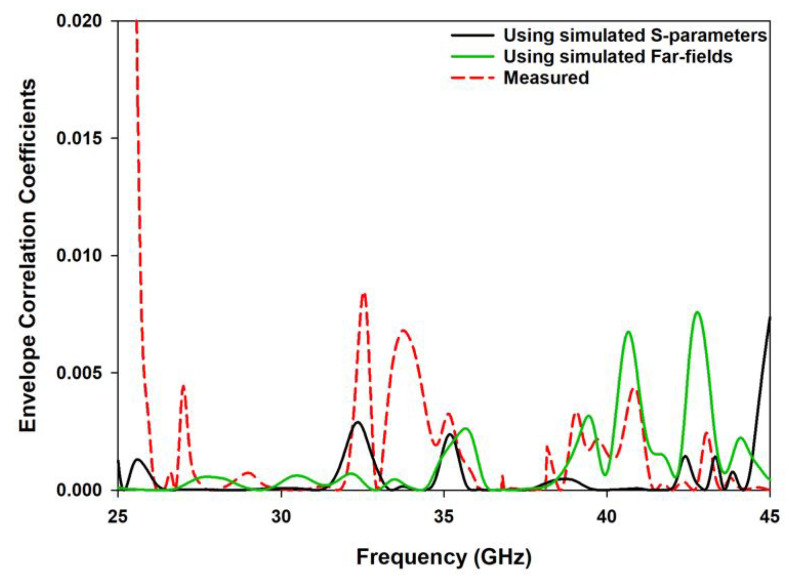
The simulated and measured ECC of the proposed MIMO antenna.

The diversity gain and other key parameters of the MIMO antennas are defined in terms of the envelope correlation coefficient (ECC). The isotropic envelope correlation coefficients were computed for the frequency bands of operation as illustrated in Figure 11. The calculated values are limited to 0.02, which demonstrates the suitability of the proposed antenna for wireless communication applications. The ECC of the dual MIMO antenna was computed by utilizing the following Equation [31].

(5) $$\rho_{e}=\frac{{\left|S_{11}^{*}S_{12}+S_{21}^{*}S_{22}\right|}^{2}}{\left(1-{\left|S_{11}\right|}^{2}-{\left|S_{21}\right|}^{2}\right)\left(1-{\left|S_{22}\right|}^{2}-{\left|S_{12}\right|}^{2}\right)}$$

### 4.2. Analysis of Diversity Gain

**Figure 12:**
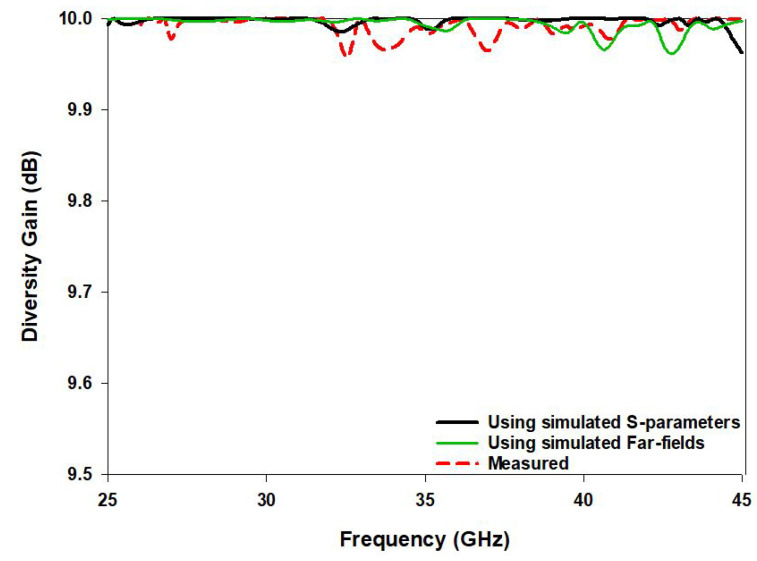
The simulated and measured diversity gain of the proposed MIMO antenna.

The diversity gain for the frequency bands of operation has been determined from the far field and S parameters as illustrated in Figure 12. This parameter is calculated based on the envelope correlation coefficient, as [32],

(6) $$DG=10\sqrt{1-{\left|\rho_{e}\right|}^{2}}$$

The simulated and measured diversity gains over the entire frequency band of operation are 9.98 and 9.97, respectively. The measured values are slightly lower than the simulated values due to fabrication and experimental tolerances.

### 4.3. Channel Capacity Loss (CCL)

**Figure 13:**
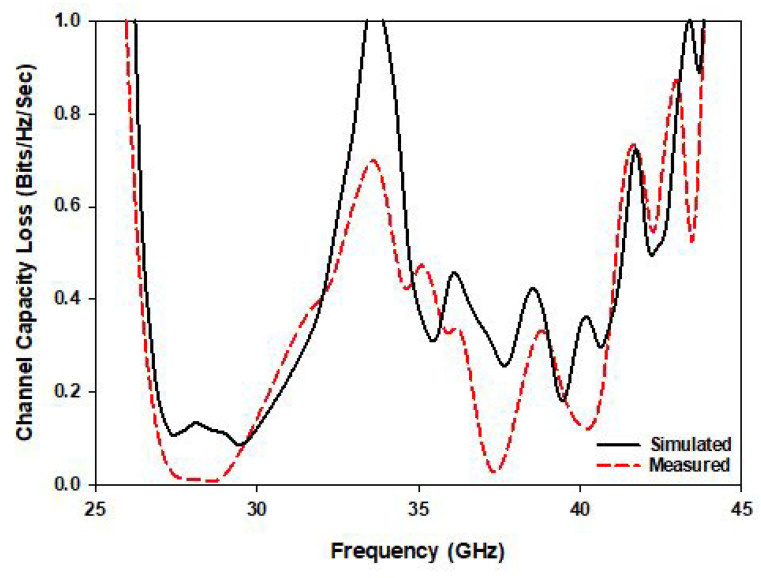
The simulated and measured CCL of the proposed MIMO antenna.

The CCL of an antenna indicates the quality of the transmitted data over the frequency band of operation. A high data transfer rate can be obtained for lower CCL. For example, a CCL higher than 0.4 bits/s/Hz indicates lossy data transmission while excellent transmission is assumed otherwise. As demonstrated in Figure 13, the calculated and measured CCLs are lower than 0.4 bits/s/Hz at the desired frequency bands. In detail, the CCL is equal to 0.3 and 0.1 at the lower and higher frequency bands, respectively. Accordingly, since the achieved CCL at both bands is ≤0.4 bits/s/Hz, the proposed MIMO antenna offers an efficient data transmission with low loss [33]:

(7) $$\begin{array}{c} C_{loss}=-\log_{2}\det\left(\psi \right) \end{array}$$

where ψ is the correlation matrix,

(8) $$\begin{array}{c} \Psi =\left|\begin{array}{cc} \psi_{11} & \psi_{12}\\ \psi_{21} & \psi_{22} \end{array}\right|\\ \Psi_{11}=1-\left({\left|S_{11}\right|}^{2}+{\left|S_{12}\right|}^{2}\right)\\ \Psi_{22}=1-\left({\left|S_{21}\right|}^{2}+{\left|S_{22}\right|}^{2}\right)\\ \Psi_{12}=-\left(\left(S_{11}^{*}S_{12}\right)+\left(S_{21}^{*}S_{22}\right)\right)\\ \Psi_{21}=-\left(\left(S_{22}^{*}S_{21}\right)+\left(S_{12}^{*}S_{11}\right)\right) \end{array}$$

### 4.4. Total Active Reflection Coefficient

**Figure 14:**
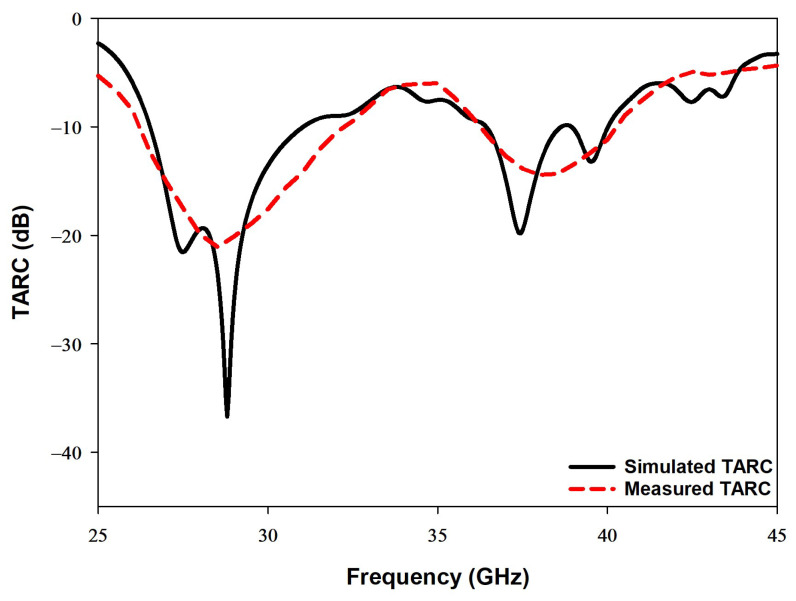
The simulated and measured TARC of the proposed MIMO antenna.

The total active reflection coefficient (TARC), as shown in Figure 14, illustrates the simulated and measured MIMO antenna systems’ effective performances, namely the operating bandwidth. Equation (9) has been utilized, which explains how the S-parameters can be used to calculate the TARC, whereby θ represents the input feeding phase [34].

(9) $$\Gamma_{a}^{t}=\sqrt{\frac{\left({\left|s_{11}+s_{12}e^{j\theta }\right|}^{2}\right)+\left({\left|s_{21}+S_{22}e^{j\theta }\right|}^{2}\right)}{2}}$$

### 4.5. The Mean Effective Gain

The mean effective gain (MEG) is another key parameter that is commonly used to characterize the performance of the MIMO antenna systems. In fading environments, the MEG measures the performance of the antenna system. Equation (10) describes how the S-parameters of a MIMO antenna system can be used to calculate MEG [35].

(10) $${\mathrm{MEG}}_{i}=0.5\eta_{{}_{i,rad}}=0.51-\left[\sum_{j=1}^{M}{\left|S_{ij}\right|}^{2}\right]$$

**Figure 15:**
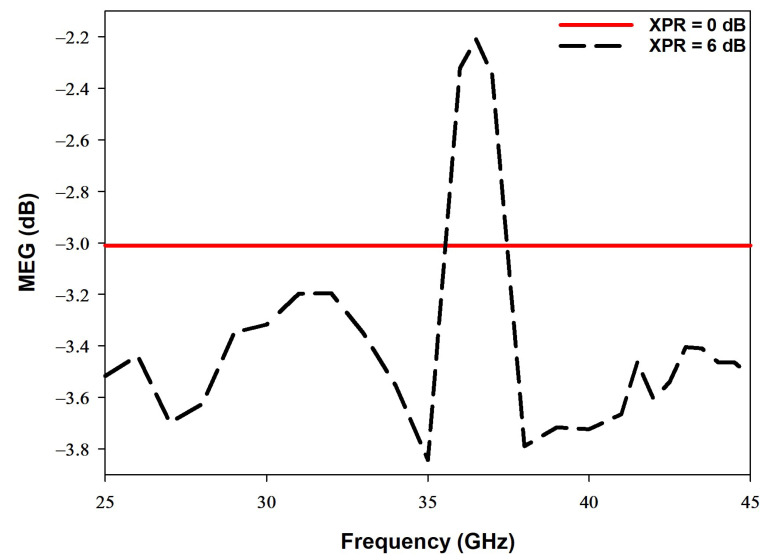
The mean effective gain (MEG) of the proposed MIMO antenna.

In the above equation, $\eta_{i}{,}_{rad},M$, and *i* represent the radiation efficiency, number of antenna elements, and the antenna under observation in the MIMO system, respectively. In order to attain practical results that can be validated, as shown in Figure 15, the MEG value should be between −3 dB and −12 dB [35]. It should be noted that XPR in Figure 15 denotes the cross-polarization power ratio where 0 dB and 6 dB refer to different mediums.

### 4.6. Multiplexing Efficiency

The multiplexing efficiency ($\eta_{\mathrm{mux}}$) is defined as the signal-to-noise ratio between imperfect and an ideal MIMO antenna systems. Equation (11) demonstrates how $\eta_{\mathrm{mux}}$ can be calculated [36].

(11) $$\eta_{\mathrm{mux}}=\sqrt{\eta_{i}\eta_{j}{\left(1-\left|\rho_{c}\right|\right)}^{2}}$$

**Figure 16:**
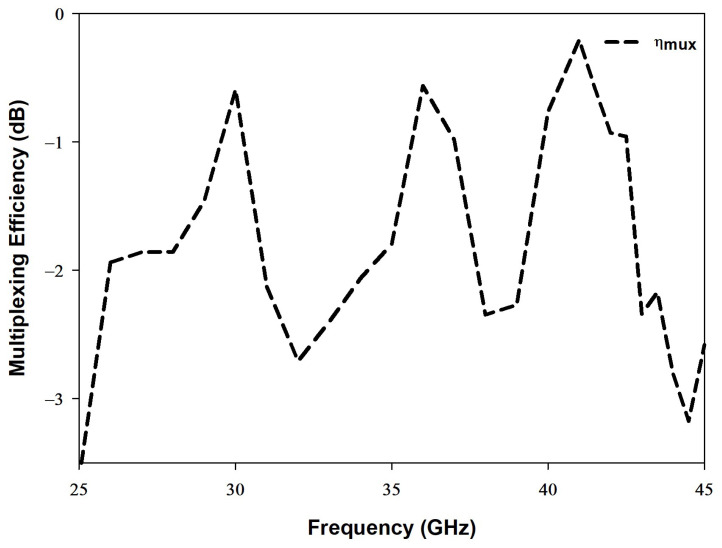
The multiplexing efficiency of the proposed MIMO antenna.

The parameters, $\eta_{i},\eta_{j}$ and $\rho_{c}$ represent total efficiency, antenna port *i* or *j*, and complex correlation coefficient, respectively. As demonstrated in Figure 16, the multiplexing efficiency should not be lower than −3 dB at the operating dual frequencies of 28 GHz and 38 GHz.

### 4.7. Comparison with Published MIMO DRA Designs

The performance of the proposed dual band mm-wave MIMO antenna has been evaluated and compared with recently published counterpart antennas as demonstrated in Table 2. As per the observations, the proposed antenna resonates at dual bands while all the reported MIMO DRAs operate in a single band. The data demonstrate that the proposed design offers the highest measured reduction in $S_{21}$ over the dual frequency bands. Further, the achieved ECC in both frequency bands is very close to the highest value reported in [15,18]. In addition, the proposed configuration offers the lowest profile of 0.11$\lambda_{0}$ compared to the MIMO DRAs reported in the literature.

Compared to published counterparts, the proposed antenna is advantageous on various aspects such as the compact size, dual band operation, and simple low-cost structure in which there is no need to incorporate additional components to improve the isolation between the two DRAs. In addition, it offers excellent performance at all the MIMO figures of merit across the bandwidths of the two considered operating frequency bands.

## 5. Conclusions

A novel MIMO rectangular DRA configuration has been proposed. The design operates at a dual band, whereby the lower band is centered at 28 GHz and the higher band is centred at 38 GHz. It is worth mentioning that the main contribution is to modify the dual band DRA arrangements in order to reduce the coupling between antennas without affecting other key parameters, such as the gain, impedance bandwidth, efficiency, and radiation pattern. This is particularly important for a mm-wave antenna design for which the performance can be easily affected by any changes in the size as well as the addition of air holes or layers. Moreover, the proposed configuration reduces the transmission coefficient in a cost-effective approach. The optimum configuration of two DRAs on opposite sides of the Rogers substrate offers the minimum measured $S_{21}$ of −41 dB and −44 dB at 28 GHz and 38 GHz, respectively. A significant diversity performance was demonstrated in terms of the ECC, DG, and CCL, which implies that the proposed antenna is most suitable for MIMO design. Owing to the dual band operation, the proposed configuration can be used in various applications, such as 5G mobile handsets. The proposed design principle has been demonstrated by utilizing two DRAs, but it is applicable for MIMO DRA systems with a higher number of elements.

## Figures and Tables

**Table 1:** Dimensions of proposed MIMO antenna (unit: mm).

*w*	*d*	*h*	*x*	*y*	$w_{c}$
4	4	1	25	15	0.5
*s*	*t*	$l_{s}$	$w_{r}$	$l_{r}$	$l_{c}$
0.28	0.06	0.1	0.06	2.3	2.4

**Table 2:** Comparison the similar designs with proposed MIMO antenna.

Ref.	Number of Elements	Height (mm)	Operating Bands (GHz)	Average Reduction in $S_{21}$ (dB)	ECC	DG	CLL
[9]	4	0.15 $\lambda_{0}$	28	14	0.0005	-	0.6
[10]	2	0.24 $\lambda_{0}$	28	12	0.013	9.9	-
[15]	2	0.24 $\lambda_{0}$	60	19	<5 × 10${}^{-6}$	-	-
[17]	2	0.2 $\lambda_{0}$	60	22	-	-	-
[18]	2	0.25 $\lambda_{0}$	60	16	<0.1 × 10${}^{-6}$	-	-
[21]	2	0.13 $\lambda_{0}$	28	22.7	-	-	-
This work	2	0.09/0.12 $\lambda_{0}$	28/38	25/27	0.007/0.003	9.98/9.99	0.06/0.09

## Data Availability

Not applicable.
